# Catalase activity of IgG antibodies from the sera of healthy donors and patients with schizophrenia

**DOI:** 10.1371/journal.pone.0183867

**Published:** 2017-09-25

**Authors:** Evgeny A. Ermakov, Ludmila P. Smirnova, Nikolay A. Bokhan, Arkadiy V. Semke, Svetlana A. Ivanova, Valentina N. Buneva, Georgy A. Nevinsky

**Affiliations:** 1 Institute of Chemical Biology and Fundamental Medicine, Siberian Division of Russian Academy of Sciences, Novosibirsk, Russia; 2 Novosibirsk State University, Novosibirsk, Russia; 3 Mental Health Research Institute, Russian Academy of Sciences, Tomsk, Russia; Kermanshah University of Medical Sciences, ISLAMIC REPUBLIC OF IRAN

## Abstract

We present first evidence showing that some electrophoretically homogeneous IgGs from the sera of patients with schizophrenia (36.4%) and their Fab and F(ab)_2_ fragments as well as from healthy donors (33.3%) possess catalase activity. The relative catalase activity of IgGs from the sera of individual schizophrenia patients (and healthy donors) significantly varied from patient to patient, but the activity of IgGs from healthy donors is on average 15.8-fold lower than that for schizophrenia patients. After extensive dialysis of purified IgGs against EDTA chelating metal ions, the relative catalase activity of IgGs decreases on average approximately 2.5–3.7-fold; all IgGs possess metal-dependent and independent catalase activity. The addition of external Me^2+^ ions to dialyzed and non-dialyzed IgGs leads to a significant increase in their activity. The best activator of dialyzed and non-dialyzed IgGs is Co^2+^, the activation by Cu^2+^, Mn^2+^, and Ni^2+^ ions were rare and always lower than by Co^2+^. Every IgG preparation demonstrates several individual sets of very well expressed pH optima in the pH range from 4.0 to 9.5. These data speak for the individual repertoire of catalase IgGs in every person and an extreme diversity of abzymes in their pH optima and activation by different metal ions. It is known that antioxidant enzymes such as superoxide dismutases, catalases, and glutathione peroxidases represent critical defense mechanisms preventing oxidative modifications of DNA, proteins, and lipids. Catalase activity of human IgGs could probably also play a major role in the protection of organisms from oxidative stress and toxic compounds.

## Introduction

One of the most relevant problems of modern psychiatry remains schizophrenia (SCZ); it is one of the most severe mental illness inherent to approximately 1% of the human population [1]. SCZ leads to a persistent violation of social adaptation, and it is a progressive mental illness occurring with polymorphic symptoms. Some changes that often start developing in utero or early childhood lead to a violation of synaptic transmission, neuronal damage and severe dysfunction [2–6]. Difficulties in the creation of new productive methods of diagnosis, treatment, and prevention of schizophrenia are associated with a lack of understanding of the molecular mechanisms of this disease.

So far, there is no unified view on the ethiopathogenesis of SCZ; there are many different theories. However, none of them at the present stage does introduce clarity. The widely-known fact is dysfunction of the glutamatergic system in SCZ [7–12]. Disbalance of dopamine-glutamate homeostasis in schizophrenia may be a possible reason of the patient's development of generalized oxidative stress [13, 14]. Also, the fact of enzymatic systems dysfunction involved in the metabolism of biogenic amines (indolamine, catecholamines) during mental disorders is known [15, 16]. Detection of neurotropic effect associated with the damages of cell membranes was postulated [17, 18]. The damage to the cell membranes of the brain may cause the formation of autoantigens and the production of auto-Abs as a consequence [19–21]. Nevertheless, the importance of immunological changes leading to the loss of the tolerance to self-antigens in the genesis of SCZ at present was not established [22]. At the same time, the MALDI mass spectra of the IgG light chains of SCZ patients are similar to those of IgGs corresponding to patients with systemic lupus erythematosus, but not to those of healthy donors [23]. DNA-hydrolyzing antibodies were found in the blood of patients with several autoimmune diseases. Wherein the blood of healthy donors or patients with diseases not leading to a significant disturbance of the immune system DNase antibodies were not found. However, Abs with DNase activity were revealed in 80% of SCZ patients. These data indicate that some schizophrenia patients may show signs of typical autoimmune processes to a certain extent [23].

One of the SCZ development reasons may be the disturbance in the neurotransmitter system functioning, associated with the change of neurotransmitter synthesis or breakdown and possible modifications of relevant receptors structure. A dysregulation of the nervous and immune systems was observed in SCZ, which can lead to changes in brain structure [24]. SCZ is not usually attributed to autoimmune diseases, despite that dysregulation of the immune system and immune cells including autoimmune processes in schizophrenia are not excluded [25, 26] and some typical autoimmune processes are revealed [23]. Therefore, the search for possible mechanisms of schizophrenia development is undoubtedly actual.

The partially reduced oxygen species (O_2_^-^, H_2_O_2_, and OH^•^) produced as intermediates and by-products of aerobic respiration in all higher organisms and appear in bodies through exposure to ionizing radiation act as potent oxidants attacking different cellular proteins, lipids, and DNA, [27–31]. Oxidative damage of many cells is ongoing and has been considered as an important pathophysiological factor in many different diseases development, including such socially significant as carcinogenesis, aging, multiple sclerosis (MS) and schizophrenia. It is believed that MS and schizophrenia have different pathogenetic mechanisms. MS is a chronic neurodegenerative disease of autoimmune nature, leading to structural damage of the myelin sheath of nerve fibres, while schizophrenia has neurotransmitter nature. However, it was convincingly demonstrated that activation of oxidative stress is a major factor in the MS and schizophrenia [32–38]. The impairment of cell metabolism, associated with changes in the activity of enzymes, including the antioxidant enzymes belonging to the class of oxidoreductases in the case of schizophrenia was shown [33, 36].

Artificial abzymes (Abzs) against chemically stable analogs of transition states of chemical reactions catalyzing more than 100 different chemical reactions have been obtained (reviewed in [39]; and refs therein). Natural IgGs and IgMs, IgAs hydrolyzing RNA, DNA, peptides, proteins, and polysaccharides were detected in the sera of patients with several autoimmune and viral pathologies (reviewed in [39–45]). Abzymes hydrolyzing peptides, proteins [46,47], and polysaccharides [48] with some exceptions in healthy volunteers are usually absent, or their activities are extremely low often at the limit of the detection methods sensitivity [40–45]. It was shown that healthy human Abs exhibit high level promiscuous, amyloid-directed, and superantigen-directed activities and autoantigen-directed and microbe-directed specificities [49, 50].

Polyclonal IgGs against different antigens from the sera of autoimmune patients are usually very heterogeneous in their affinity for specific antigens and can be separated into many subfractions by chromatography on antigen-Sepharoses [51–54]. Pools of polyclonal abzymes can contain different proportions of light chains of κ- and λ-types, Abs demonstrating different pH optima, having different net charges, metal-independent or activated by different metal ions, and characterized by different substrate affinities and specificities [39–45]. It was shown that small fractions of IgGs of all four subclasses (IgG1–IgG4) from autoimmune patients are catalytically active in the hydrolysis of different substrates [39–45]. For analysis of myelin basic protein- and DNA-hydrolyzing activities of monoclonal light chains (MLChs) corresponding to SLE phagemid library of kappa MLChs were used [55–59] (for review see [60, 61]). It was shown, that some hundreds of different monoclonal light chains hydrolyze DNA and other ones cleavage myelin basic protein; all MLChs demonstrated very different physico-chemical and enzymatic properties [55–59, 60, 61]. It should be assumed that the extraordinary diversity of these monoclonal light chains is mainly due to the significant differences in their variable regions responsible for substrate specificity and catalysis [55–59]. Heterogeneity is also observed in catalytic antibody kappa light chains and it was shown that structural diversity (heterogeneity) may exist due to the constant region domain and specific role of metal ions of the catalytic light chains [62, 63].

Different canonical enzymes protect various organisms from oxidative stress. Plant, bacterial and mammalian peroxidases, oxidoreductases, oxidases, and dismutases are mostly metal ions-dependent enzymes [31, 64–67]. Two superoxide dismutases in mammals are Mn- and Cu,Zn dependent, while in some bacteria Ni- and Fe-dependent enzymes are found [64, 65, 67]. Human catalase is Fe^2+^-dependent enzyme converting hydrogen peroxide to H_2_O and O_2_. Metal ions with variable valence (more often: Fe^2+^, Cu^2+^, and Mn^2+^) participate in electron transfer in oxidation-reduction reactions, catalyzed by enzymes [67]. Some metals with a constant valence can play an auxiliary role. For example, in the case of Cu,Zn-dependent superoxide dismutases Zn^2+^ does not directly participate in the reaction of dismutation and has a structural role, providing protein specific conformation necessary for the active enzyme center.

The highest level of catalase is found in the cytoplasm of erythrocyte and inside mitochondrial membranes. Glutathione peroxidase is a selenium-dependent enzyme (not metal ions-dependent) catalyzing the reduction of different hydroperoxides including H_2_O_2_ in the presence of glutathione [31, 64, 65, 67]. All mammalian enzymes listed above are present mostly in various cells, but in the case of superoxide dismutase, catalase, and glutathione peroxidase their low enzymatic activities can be revealed in the blood serum, it decreases in the blood with high speed [66, 67].

A comparison of catalase, superoxide dismutase, H_2_O_2_-dependent peroxidase, and H_2_O_2_ independent oxidoreductase activities of polyclonal IgGs obtained from the sera of healthy Wistar rats have been carried out [68]. Approximately 83% of IgGs possess superoxide dismutase activity, while all preparations oxidized 3,3'-diaminobenzidine in the presence and the absence of hydrogen peroxide. It was shown that only 17% of IgG preparations possess catalase activity. It has been demonstrated that comparable peroxidase activities are possessed by IgGs from the serum of acute viral hepatitis B or C patients as well as healthy donors [69, 70].

It was shown that small fractions of electrophoretically homogeneous IgGs and their Fab and F(ab)_2_ fragments from the sera of healthy humans oxidize 3,3'-diaminobenzidine in the presence of H_2_O_2_ through a peroxidase activity and in the absence of H_2_O_2_ through an oxidoreductase activity [71]. At present, there is no available data concerning catalase activity of Abs of healthy donors and patients with any diseases.

In this report, we have shown the first evidence of catalase activity of polyclonal IgGs isolated from the sera patients with schizophrenia and healthy donors.

## Results

### Purification and characteristics of IgGs

In this work, the sequential chromatography of the serum proteins on Protein G-Sepharose, followed by gel filtration in acidic conditions destroying immune complexes as in [71, 72] was used for isolation of immunologically and electrophoretically homogeneous polyclonal IgGs from the sera of 21 healthy donors and 22 schizophrenia patients. It was shown previously, that this approach of IgGs purification provides IgGs containing no any admixtures of proteins or canonical enzymes [40–45].

**Fig 1:**
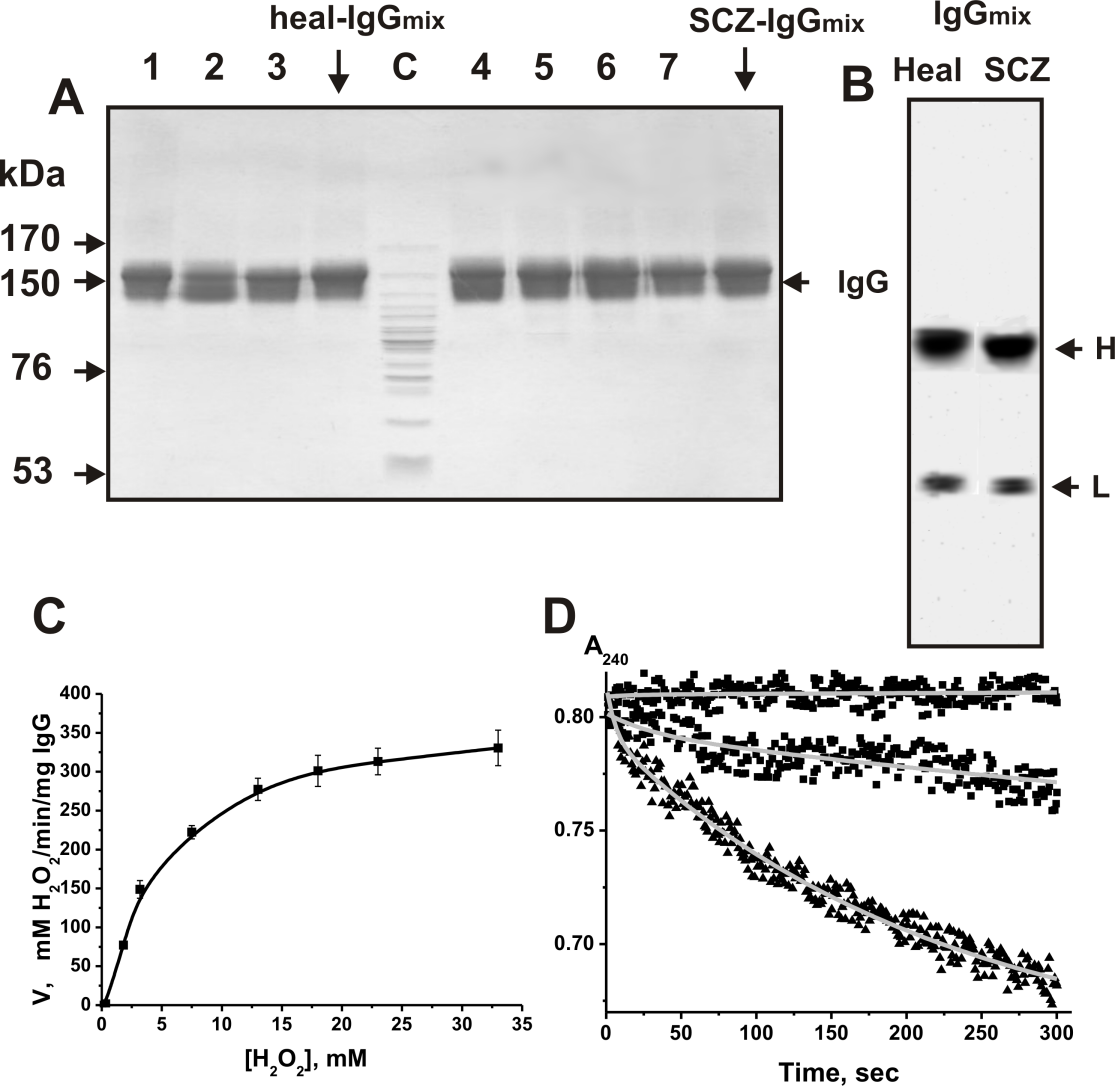
**SDS-PAGE analysis of individual IgG**_**s**_ **(10 μg) corresponding to three individual healthy donors** (lanes 1–3) and IgG_mix_ (lane healthy-IgG_mix_) as well as to four individual schizophrenia patients (lanes 4–7) and scz-IgG_mix_ (lane scz-IgG_mix_) in a nonreducing 3–16% gradient gel (A) or IgG_mix_ using a reducing 12% gel (B) followed by silver staining. Before analysis of IgG_mix_ antibodies in a reducing gel, they were boiled in the presence of DTT. The positions of standard molecular mass markers (lane C) are indicated with the arrows. Typical dependency of the relative activity of IgG(7) on the concentration of H_2_O_2_ (D)_._ Typical time-dependencies of the decrease in 30 mM H_2_O_2_ absorbance at 240 nm (A_240_) in the presence of 200 nM IgG(1) and IgG(11) as well as 2 nM IgG(8) (D) corresponding to different individual patients. For details, see Materials and methods.

To analyze an “average” situation concerning homogeneity of IgGs, we have prepared a mixture of equal amounts of polyclonal IgGs (scz-IgG_mix_) from the sera of 22 schizophrenia patients and 21 healthy donors (healthy-IgG_mix_). SDS-PAGE confirmed the homogeneity of all typical 150-kDa individual IgGs as well as healthy-IgG_mix_ and scz-IgG_mix_. Silver staining (for example, Fig 1A) of SDS-PAGE gel under nonreducing conditions showed a single band and after reduction—two bands corresponding to the H and L chains (Fig 1B).

It was shown, that IgG subfractions with maximal affinity to different metal ions do not loose intrinsically bound metals during their standard purification [73–75]. First, we analyzed the relative activity (RA) of IgGs before dialysis against EDTA and in the absence of external metal ions.

It was shown, that canonical catalases do not obey the conventional Michaelis-Menten kinetics and at high H_2_O_2_ concentration (< 10 mM): the inhibition of enzymes due to their suicidal inactivation was observed [76, 77]. For all catalytically active antibodies, the relationships between the rate and the concentration of H_2_O_2_ were found to be close to those for the Michaelis-Menten dependences and at a concentration of 30 mM hydrogen peroxide these dependences were close to the plateau (Fig 1C). Similar situation was observed earlier for rat IgGs with peroxidase and catalase activities [71, 73–75] and human Abs with peroxidase activities [72].

It was shown that 6 of 22 scz-IgG preparations possess high catalase activity. Fig 1D demonstrates typical kinetic curves corresponding to the decrease in H_2_O_2_ absorbance at 240 nm (A_240_) in the presence of IgG(1), IgG(8), and IgG(11). Also, by similar way, it was shown, that 7 of 21 healthy-IgG preparations also possess catalase activity.

### Application of the strict criteria

**Fig 2:**
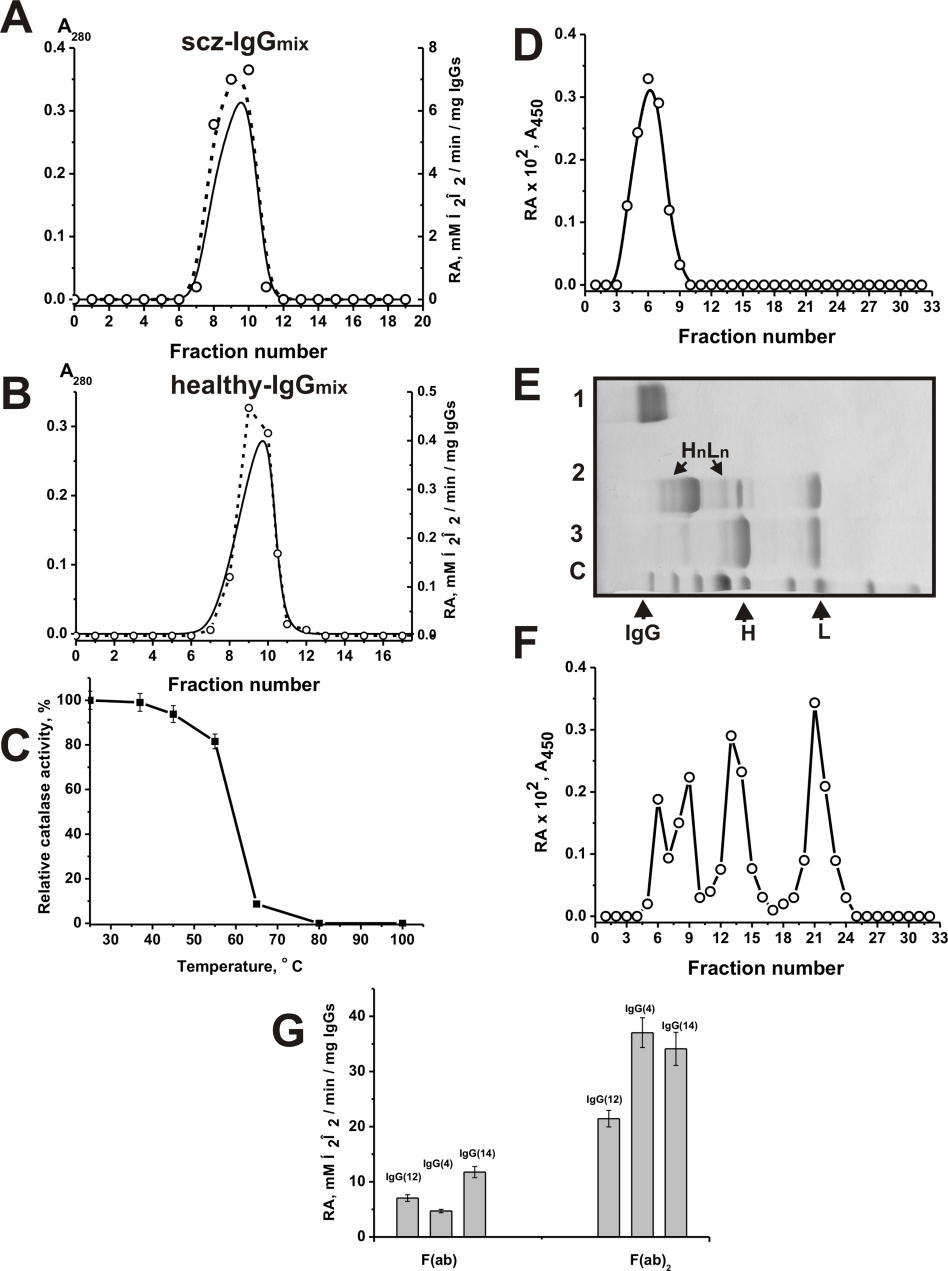
Strict criteria prove that the catalase activity is intrinsic properties of scz-IgG_mix_ and health-IgG_mix_. Preparations of scz-IgG_mix_ (**A**) and healthy-IgG_mix_ (**B**) were separated by FPLC gel filtration on a Superdex 200 column in an acidic buffer Gly-HCl pH 2.6 after Abs incubation in the same buffer: (—), absorbance at 280 nm (A_280_); (○), relative activity (RA) of the IgG_mix_ in the degradation of H_2_O_2_. Analysis of the thermal stability of scz-IgG_mix_; the preparation was preincubated at various temperatures for 10 minutes and then its relative catalytic activity was estimated using standard conditions (C). SDS-PAGE analysis of catalase activity of intact scz-IgG_mix_ (**D**) as well as separated H, L chains and their L_n_H_n_ oligomers (**F**) in non-reducing SDS-PAGE gradient 4–15% scz-IgG_mix_ before (**D**) and after treatment of IgGs with DTT (**F**); panel **F** corresponds to lane 2 of Panel **E**. The relative catalase activity (RA, A_240_/min) was revealed using the extracts of 2-3-mm many fragments of one longitudinal slice of the gel corresponding IgGs before (**D**) and after treatment with DTT (**F**). The control longitudinal slices of the same gels were stained with Coomassie R250 (**E**): lane 1 corresponds to intact IgG_mix_, lane 2 to IgG_mix_ incubated with 40 mM DTT for 10 min at 30°C, lane 3 to IgG_mix_ boiled with DTT. Lane C (**E**) shows the positions of molecular mass standard markers. The relative activity of F(ab) and F(ab)_2_ fragments of individual IgG(4), IgG(12), and IgG(14) (**G**). The average error of the initial rate determination from two experiments did not exceed 10–15%. For details, see Materials and methods.

We have applied several previously developed strict criteria [40–46] proving that the catalase activity of IgGs of the sera schizophrenia patients and healthy donors belongs to the Abs and is not due to co-purifying enzymes. The criteria may be summarized as a) the IgG_mix_ (corresponding to the central parts of the peaks after gel filtration) was electrophoretically homogeneous (Fig 1A); b) gel filtration of scz-IgG_mix_ (Fig 2A) and healthy-IgG_mix_ (Fig 2B) under conditions dissociating strong noncovalent complexes in an acidic buffer (pH 2.6) did not eliminate the catalase activity, and the peaks of the activity tracked exactly with the intact IgGs; c) immobilized mouse polyclonal IgGs against the light chains of human IgGs completely absorbed the catalase activity (data nor shown); d) Fab and F(ab)_2_ fragments of scz-IgG_mix_ demonstrate catalase activity (Fig 2G).

IgGs of SCZ patients were analyzed in more detail. The denaturation of IgGs was analyzed by different circular dichroism spectroscopy and calorimetric methods [78]. It was shown, that a significant denaturation of monoclonal mouse Abs begins at approximately 61°C. Unpredictable high thermostability recombinant antibody L chain-against porphyrin Fe(III) complex with peroxidase activity demonstrating high optimal reaction temperature (90°C) was described [79]. As shown by us on the example of human abzymes with nuclease and some other enzymatic activities, they almost completely lose their activity after incubation for 10 min even at 60–80° C [80, 81]. Polyclonal IgGs with catalase activity also demonstrated low termostability and they lose the activity after incubation at 60 and especially 80–100° C (Fig 2C).

To exclude artifacts of possible contamination of co-isolating enzymes, the intact scz-IgG_mix_ preparation was also separated by SDS-PAGE, and its catalase activity was detected after extraction of proteins from the separated gel slices (Fig 2D, the position of intact IgGs is shown in Fig 2E, lane 1). The SDS-PAGE assay showed the absence of catalase activity in scz-IgG_mix_ after boiling of SCZ IgG under reducing conditions and in separated L or H-chains (Fig 2F, positions of the separated chains after IgG boiling are shown in Fig 2E, lane 3). However, SDS-PAGE revealed catalase activity of scz-IgG_mix_ after it mild treatment with buffer containing 40 mM DTT and 0.1% SDS (37°C, 10 min) in the bands corresponding to the separated L and H chains and its partially reduced complexes (Fig 2F catalytic activity; positions of the separated chains of partially reduced IgG_mix_ are shown in Fig 2E, lane 2).

It should be mentioned that after a mild treatment with DTT there was no complete dissociation of IgGs for free light and heavy chains (Fig 2E, lane 2). Catalase activity was revealed in the bands corresponding to the different partially reduced H_n_L_n_ forms of IgGs (Fig 2F). Interestingly, similar results were observed earlier for several other IgGs with oxidoreductase activities. Separated light and heavy chains and different partially reduced H_n_L_n_ forms of rat [75] and human [71] IgGs were active in the 3,3'-diaminobenzidine oxidation by a peroxidase activity in the presence of H_2_O_2_ and also an oxidoreductase activity in the absence of H_2_O_2_.

Any protein complexes dissociate in the presence of SDS, and the electrophoretic mobility of canonical catalases cannot coincide with intact and reduced IgGs. Since that the detection of catalase activity in the gel regions corresponding only to intact IgGs (150 kDa) before reduction and in the slices corresponding to the light, heavy chains, and H_n_L_n_ combinations after mild reduction, together with the absence of any other bands of the activity or protein (Fig 2), provides direct evidence that scz-IgGs and healthy-IgGs can possess catalase activity. Several other strict criteria were also fulfilled (see below).

### Estimation of the relative catalase activity

We have shown that the catalase activity is an intrinsic property of schizophrenia patients IgG (see above) and that the Abs purified on Protein G-Sepharose and by gel filtration do not contain any catalytic impurities, and their relative activity can be evaluated without additional purification. To estimate the catalase activity quantitatively, we have found the concentration for each IgG preparation for conversion of H_2_O_2_ to water and O_2_ corresponding to conditions of the pseudo-first order reaction within linear regions of Ab concentration and time curves. The relative specific activities of IgGs from the sera of 22 individual SCZ patients significantly varied from patient to patient (range (0–13.7)×10^3^ specific units (SU; mM H_2_O_2_/min/mg IgGs), but 8 of 22 samples (36.4%) had high catalase activity (Table 1). The RAs for individual IgGs do not correspond to normal Gaussian distribution. We have estimated the median (M) and interquartile ranges (IQR). The median of RAs for SCZ antibodies was zero (M = 0.0 SU, IQR; Q1 = 0 and Q2 = 3799.6 SU) and significantly different in comparison with the average RA value ((1.9±2.6)×10^3^ SU) (Table 1). The apparent *k*_*cat*_ values at the fixed concentration of H_2_O_2_ (30 mM) for 22 scz-IgGs with catalase activity were calculated (range 0–2.6×10^5^ min^-1^, average value (2.8 ± 3.7 ×10^4^ min^-1^, Table 1). Also, the average RAs for eight individual scz-IgGs demonstrating catalase activity was calculated, (5.2 ± 3.0)×10^3^ SU (*k*_cat_ = 7.6 ± 4.6×10^4^ min^-1^) (Table 1).

**Table 1:** The relative activity and the apparent *k*_*cat*_ values characterizing degradation of H_2_O_2_ by individual IgGs from the sera of schizophrenia patients.

Number of patient	Specific activity× 10^−3^, mM H_2O_O_2_/min/mg IgGs	apparent *k*_*cat*_×10^−4^, min-1
1	0^μ^	0
2	0	0
3	0	0
4	6.7*	10.0**
5	4.1	6.2
6	0	0
7	2.2	3.3
8	13.7	20.6
9	0	0
10	0	0
11	0.32	0.48
12	3.8	5.7
13	0	0
14	7.1	10.7
15	0	0
16	0	0
17	0	0
18	0	0
19	0	0
20	0	0
21	3.8	3.7
22	0	0
Average value for 22 IgGs	1.9±2.6	2.8±3.7
Median (IQR***)	0.0 (Q1 = 0 and Q2 = 3.8)	0.0 (Q1 = 0 and Q2 = 3.7)
Average value for 8 active IgGs	5.2±3.0	7.6±4.6
Median (IQR)	3.95 (Q1 = 3.0 and Q2 = 6.9)	5.95 (Q1 = 3.5 and Q2 = 10.35)

*For each value, a mean of three measurements is reported; the error of the determination of values did not exceed 7–15%.

**The apparen_*t*_ values of kcat reaction at fixed concentration of H_2_O_2_ (30 mM) were calculated using average relative activity values: *k*_*cat*_ *=* V (M/min)/ [IgGs] (M)

***IQR is interquartile ranges

^μ^Zero means the absence activity of the antibody in the absence and in the presence of different exogenous metal ions.

The *K*_m_ and *k*_cat_ values were determined in the case of scz-IgG(8): *K*_m_ = 19.2±1.5 mM, *k*_cat_ = (3.4±0.3)×10^5^ min^-1^. Scz-IgG(14) demonstrated lower *K*_m_ = 9.4±0.7 mM and *k*_cat_ = (1.9±0.16)×10^5^ min^-1^.

Seven of 21 individual IgGs (33.3%) from healthy donors also possess catalase activity (Table 2). The RAs for total group of 21 individual donors (range: (0–0.71)×10^3^ SU) also do not correspond to the Gaussian type of distribution and the median is 0.0 SU (IQR; Q1 = 0 and Q2 = 0.22×10^3^ SU) was significantly different comparing with the average RA value (0.12±0.17)×10^3^ SU, average *k*_cat_ = (0.17 ± 0.25)×10^4^ min^-1^ (Table 2). The average RA (0.37±0.19 SU) and apparent *k*_cat_ (0.56 ± 0.29×10^4^ min^-1^) for seven individual healthy-IgGs demonstrating catalase activity were calculated (Table 2).

**Table 2:** The relative activity and the apparent *k*_*cat*_ values characterizing degradation of H_2_O_2_ by individual IgGs from the sera of healthy donors.

Number of donor	Specific activity× 10^−3^, mM H_2_O_2_/min/mg IgGs*	Apparent *k*_*cat*_×10^−4^, min^-1^
1	0^μ^	0**
2	0.64	0.96
3	0	0
4	0	0
5	0	0
6	0.24	0.36
7	0	0
8	0.24	0.36
9	0	0
10	0	0
11	0	0
12	0.71	1.1
13	0	0
14	0	0
15	0	0
16	0.42	0.63
17	0	0
18	0	0
19	0	0
20	0.22	0.33
21	0.11	0.17
Average value for 22 IgGs	0.12±0.17	0.17±0.25
Median (IQR***)	0.0 (Q1 = 0, Q2 = 0.22)	0.0 (Q1 = 0; Q2 = 0.33
Average value for 7 active IgGs	0.37±0.19	0.56±0.29
Median (IQR)	0.24 (Q1 = 0.22, Q2 = 0.64	0.36 (Q1 = 0.36, Q2 = 0.96)

*For each value, a mean of three measurements is reported; the error of the determination of values did not exceed 7–15%.

** The values of apparent *k*_*cat*_ of the reaction at fixed concentration of H_2_O_2_ (30 mM) were calculated using average values of RA: *k*_*cat*_ *=* V (M/min)/ [IgGs] (M)

***IQR is interquartile ranges.

^μ^Zero means the absence activity of the antibody in the absence and in the presence of different exogenous metal ions.

Since only 33.3–36.4% of SCZ patients and healthy donors exhibited catalase activity, the medians in both cases were zero, and there was no statistical difference between these groups (*P* = 0.44). In spite of the absence of statistical difference between these whole groups, the relative average activity of IgGs corresponding to schizophrenia patient is approximately 15.8–fold higher than that for healthy donors. In addition, the subgroups of eight scz-IgGs (median = 5.95×10^3^ SU; Q1 = 3.5×10^3^ and Q2 = 10.35×10^3^ SU) and seven healthy-IgGs (median = 0.24×10^3^ SU; Q1 = 0.22×10^3^ and Q2 = 0.64×10^3^ SU) with catalase activity demonstrate statistical significant difference in the activities, *P* = 0.0026. Thus, the median for eight scz-IgGs is ~24.8-fold higher than that for seven healthy-IgGs.

### Determination of the kinetic parameters

**Fig 3:**
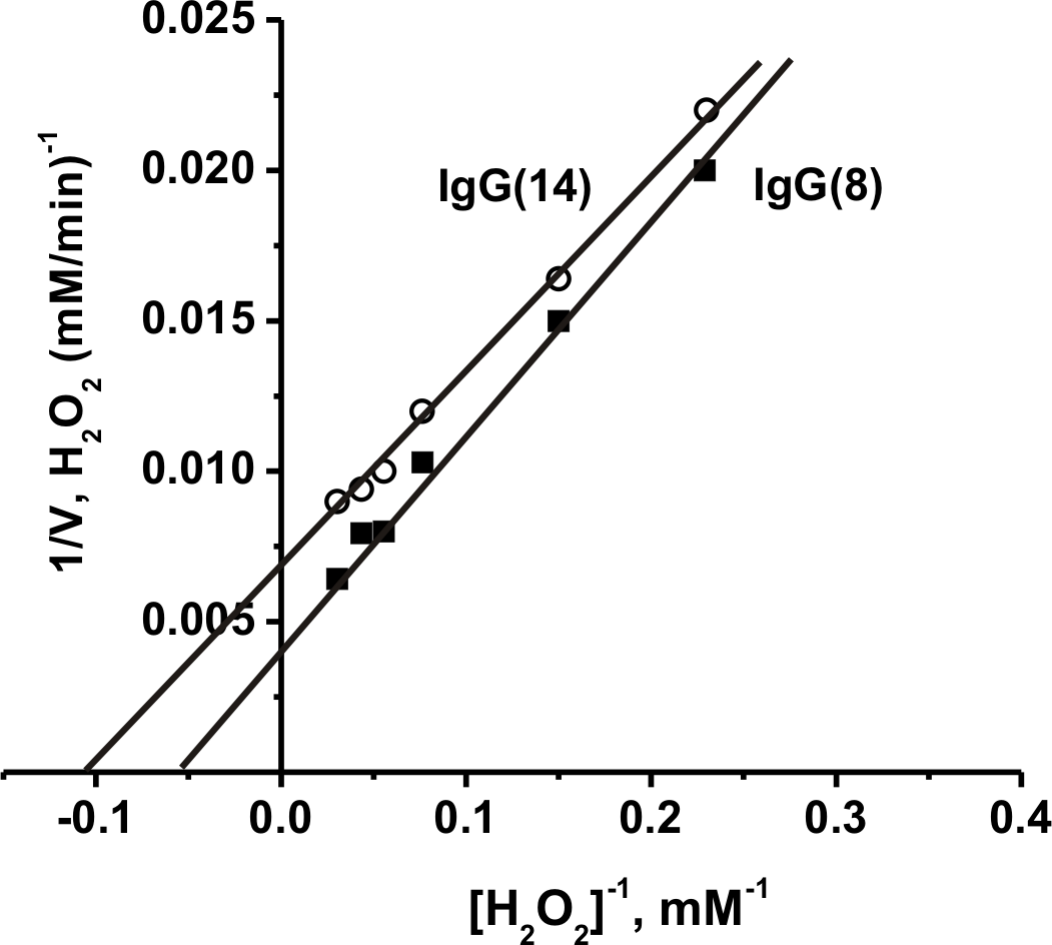
Dependencies of relative rates of catalase activity on the concentration of H_2_O_2_ and determination of the *K*_*m*_ and *V*_*max*_ (*k*_cat_) values using the Lineweaver–Burk plot in the case of scz-IgG8 (0.74 μM) and scz-IgG14 (0.72 μM). The error of the initial rate determination from three experiments in every case did not exceed 7–10%. For details, see Materials and methods.

We have estimated the *K*_m_ for H_2_O_2_, and *V*_max_ (*k*_*cat*_) values using two individual scz-IgGs. The dependencies of the initial rate on H_2_O_2_ concentration in the reaction catalyzed by scz-IgG(8) and scz-IgG(14) are consistent with Michaelis–Menten kinetics (Fig 3). The *K*_m_ and *k*_cat_ values were determined in the case of scz-IgG(8): *K*_m_ = 19.2±1.5 mM, *k*_cat_ = (3.4±0.3)×10^5^ min^-1^. Scz-IgG(14) demonstrated lower *K*_m_ = 9.4±0.7 mM and *k*_cat_ = (1.9±0.16)×10^5^ min^-1^.

### Influence of metal ions on peroxidase and oxidoreductase activities of IgGs

The well-known bacterial and eukaryotic oxidases, peroxidases, and dismutases that convert active forms of oxygen are metal-dependent enzymes. Mammalian catalase is a heme–Fe^2+^ dependent enzyme [82]. As it was mentioned above, IgGs do not lose a part of intrinsically bound metals during their standard purification. In the case of rat IgGs, removal of the intrinsically bound different Me^2+^ (metal) ions by dialysis against EDTA or addition of 0.1 M EDTA directly to the reaction mixture, antibody preparations completely lose both peroxidase and oxidoreductase activity, and the activities were regained after addition of external Fe^2+^, Cu^2+^, Mn^2+^ and other ions or different combinations [72, 74]. In contrast to rat IgGs, human IgGs did not lose peroxidase and oxidoreductase activities completely after dialysis against EDTA [71].

**Fig 4:**
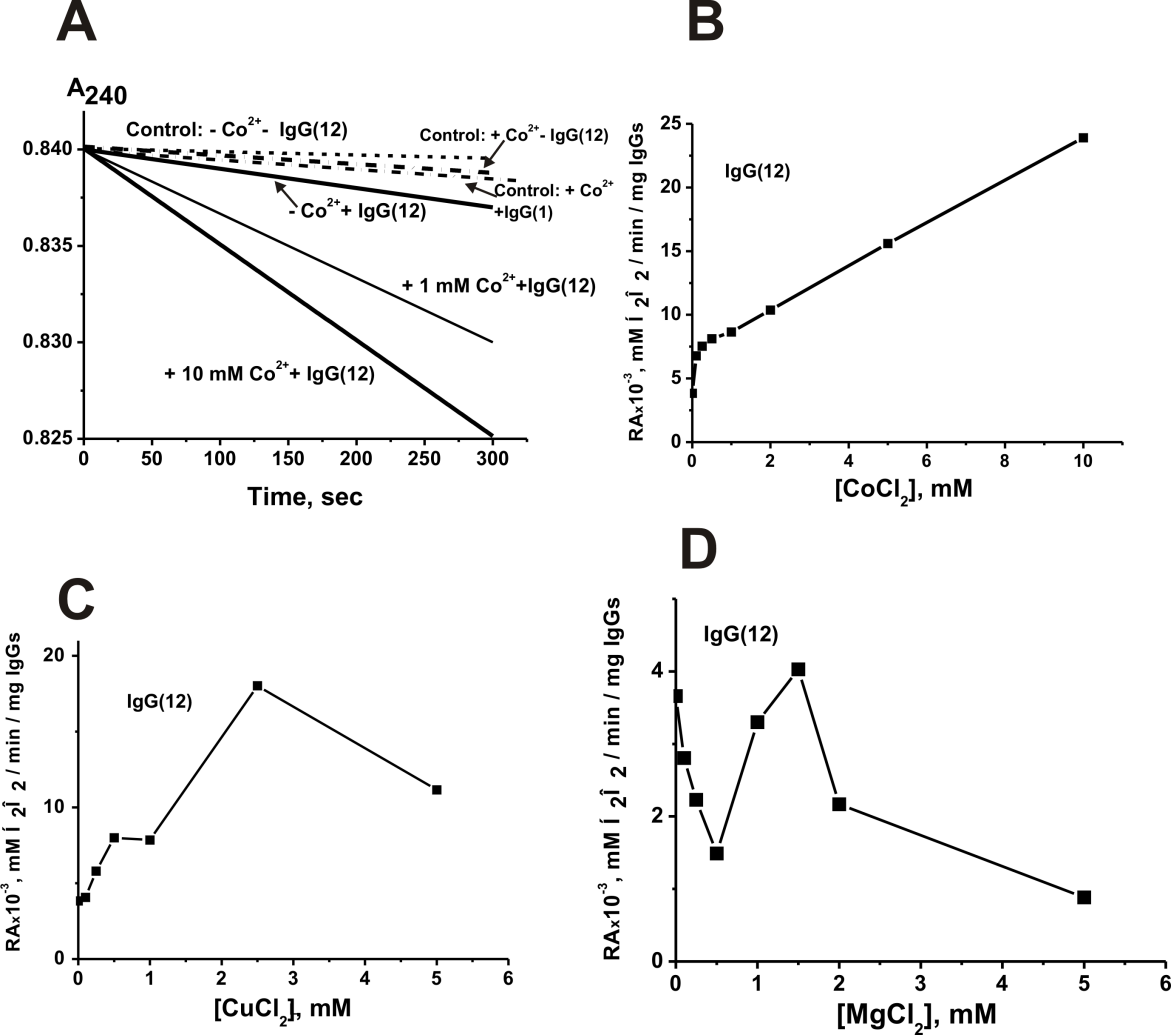
**Time-dependent changes in the decrease of A**_**240**_ **due to H**_**2**_**O**_**2**_ **degradation** in the absence and the presence of 0.3 μM IgG(12) and CoCl_2_ in different concentrations (**A**). The curves of the Panel A correspond to the averaged values of the decrease in A_240_ obtained by analogy with Fig 1C. The average curves (control:—Co^2+^—IgG) and (control: + Co^2+^—IgG) correspond to the reaction mixtures incubated in the absence and in the presence of 10 mM CoCl_2._ The dependencies of the specific activities of IgG(12) on the concentration of Co^2+^ (B), Cu^2+^ (C), and Mg^2+^ (D) ions. All SCZ IgGs, metal ions used and other details are marked on Panels A-D. For details, see Materials and methods.

We have analyzed an effect of dialysis against 0.3 M EDTA and addition of 0.1 M EDTA directly to the reaction in the case of three individual scz-IgGs (Fig 4). The catalase activity of the IgGs dependently on preparation was decreased 2.5–3.7-fold (average value 3.1 ± 0.4-fold), but all Abs were catalytically active. Similar to [71, 72, 83] we have analyzed for the metal composition of dialyzed IgG preparations by two-jet arc plasmatron atomic emission and dialyzed IgG preparations did not contain any metal ions. Therefore, we concluded that in contrast to rat IgGs, completely dependent on metal ions [72, 74], human IgGs possess metal-dependent and metal-independent catalase activity similar to such type of peroxidase and oxidoreductase activities of human IgGs [71].

It is known, that ions of metals with variable valence can stimulate degradation of H_2_O_2_. Therefore, we first analyzed time-dependencies of the decrease in A_240_ in the absence or the presence of different metal ions and scz-IgGs. It should be mentioned that there was spontaneous degradation of H_2_O_2_ (curve -Co^2+^- IgG, Fig 4A) while metal ions especially with variable valences stimulated its disproportion; for example curve +Co^2+^- IgG of Fig 4A. However, the disproportionation of H_2_O_2_ in the presence of only ions of various metals in different concentrations and also of metal ions together with IgGs without catalase activity (curve +Co^2+^+ IgG(1), Fig 4A) did not exceed 1–10% of the hydrogen peroxide decomposition in the presence of catalytically active abzymes in the presence metal ions. Non-dialyzed IgGs without catalytic activity were inactive in the presence and in the absence of different external metal ions (Tables 1 and 2).

Fig 4A demonstrates typical time-dependencies of catalase activity in the presence of CoCl_2_ in various concentrations as well as spontaneous degradation of H_2_O_2_ in the absence and the presence of 10 mM CoCl_2_. The relative catalase activity of scz-IgGs was calculated from the difference of the curves slopes corresponding to the spontaneous decomposition of the peroxide in the presence of only CoCl_2_ (in fixed concentration) and the H_2_O_2_ disproportion in the presence of IgG(12) and Co^2+^ ions in the same concentration. The dependence of IgG(12) specific activity on the concentration of CoCl_2_ was measured using the data corresponding to different fixed metal ions concentrations (for example, Fig 4B). The relative activity of other antibody preparations in the presence of different metal ions was evaluated in the same way. Several metal ions demonstrated complex dependencies with two close maxima (Fig 4C), or first were observed the inhibition and then the activation of the catalase activity (Fig 4D).

**Fig 5:**
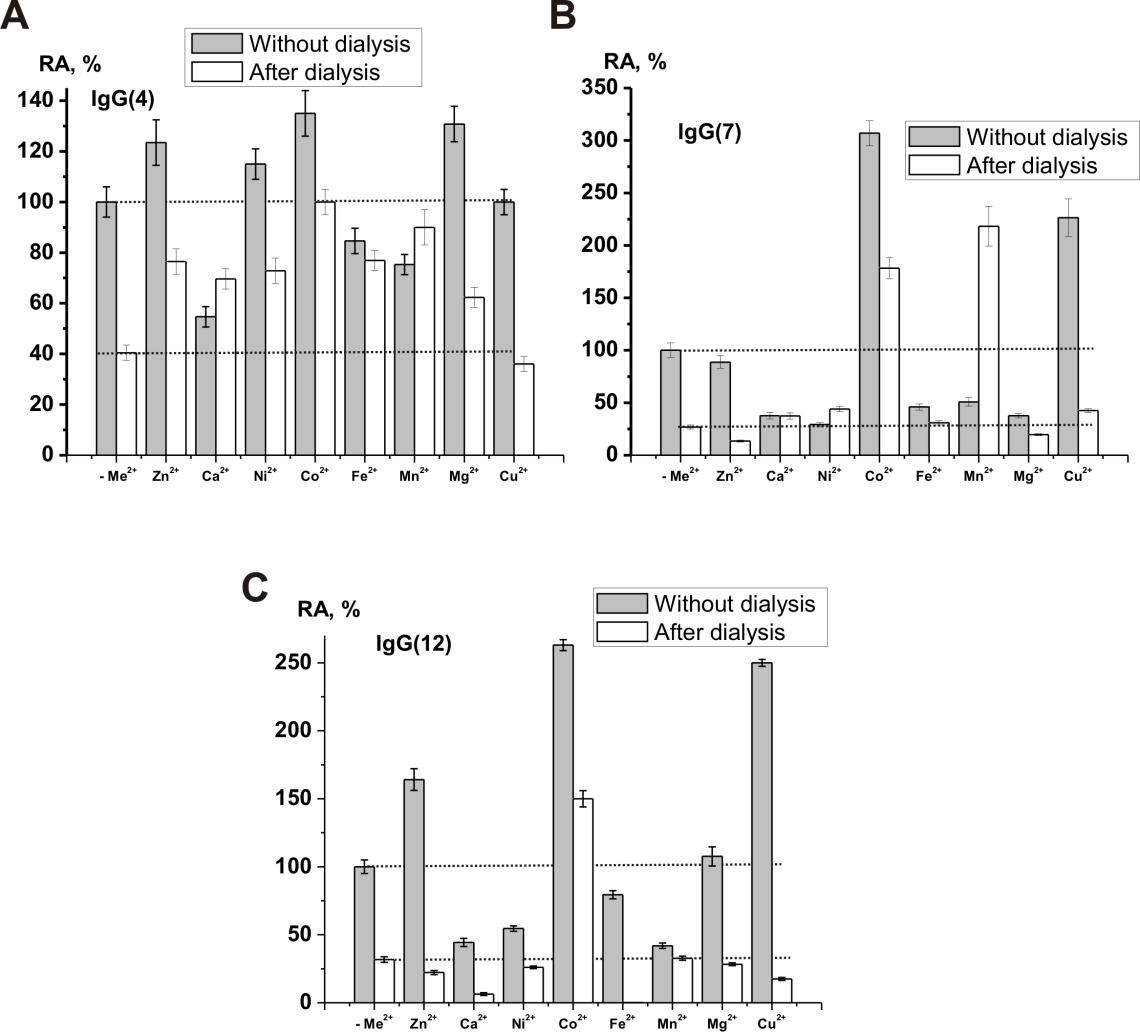
**Effect of the dialysis of IgGs against EDTA for three individual scz-IgGs** and different metal ions on the relative activity (RA, %) of dialyzed and non-dialyzed preparations (A-C). The RA of every non-dialyzed preparation was taken for 100%. All IgG preparations, metal ions, and conditions used marked on Panels A-C. For details, see Materials and methods.

It should be mentioned, that all IgGs demonstrated good activity in the presence of different metal ions at ~1.5 mM concentration. Therefore the effect of 1.5 mM different metal ions on the relative activity of three scz-IgGs before and after their dialysis against EDTA was analyzed (Fig 5). One can see, that maximal increase in the RA of non-dialyzed IgGs in the presence of external metal ions was dependent on the IgG preparation; IgG(4): Co^2+^ ≥ Mg^2+^≥ Zn^2+^≥ Ni^2+^ (Fig 5A); IgG(7): Co^2+^ > Cu^2+^(Fig 5B); IgG(12): Co^2+^ ≥ Cu^2+^ > Zn^2+^ (Fig 5C). After dialysis the IgG(4) was activated by different metal ions: Co^2+^ ≥ Mn^2+^ ≥ Fe^2+^ ≥ Zn^2+^ ≥ Ni^2+^ ≥ Ca^2+^ ≥ Mg^2+^ (Fig 5A). All metal ions except Mn^2+^ and Co^2+^ inhibited or not substantially affected catalase activity of dialyzed IgG(7) (Fig 5B). Dialyzed IgG(12) increased its activity only in the presence of Co^2+^. Thus, every of three scz-IgG preparations demonstrates specific dependencies on various metal ions before and after Abs dialysis. IgG(4) was activated by all metal ions used except Cu^2+^ before and after its dialysis. These data can speak for an individual repertoire of Me^2+^-dependent catalase IgGs in the sera of every SCZ patient.

### pH optima assay

**Fig 6:**
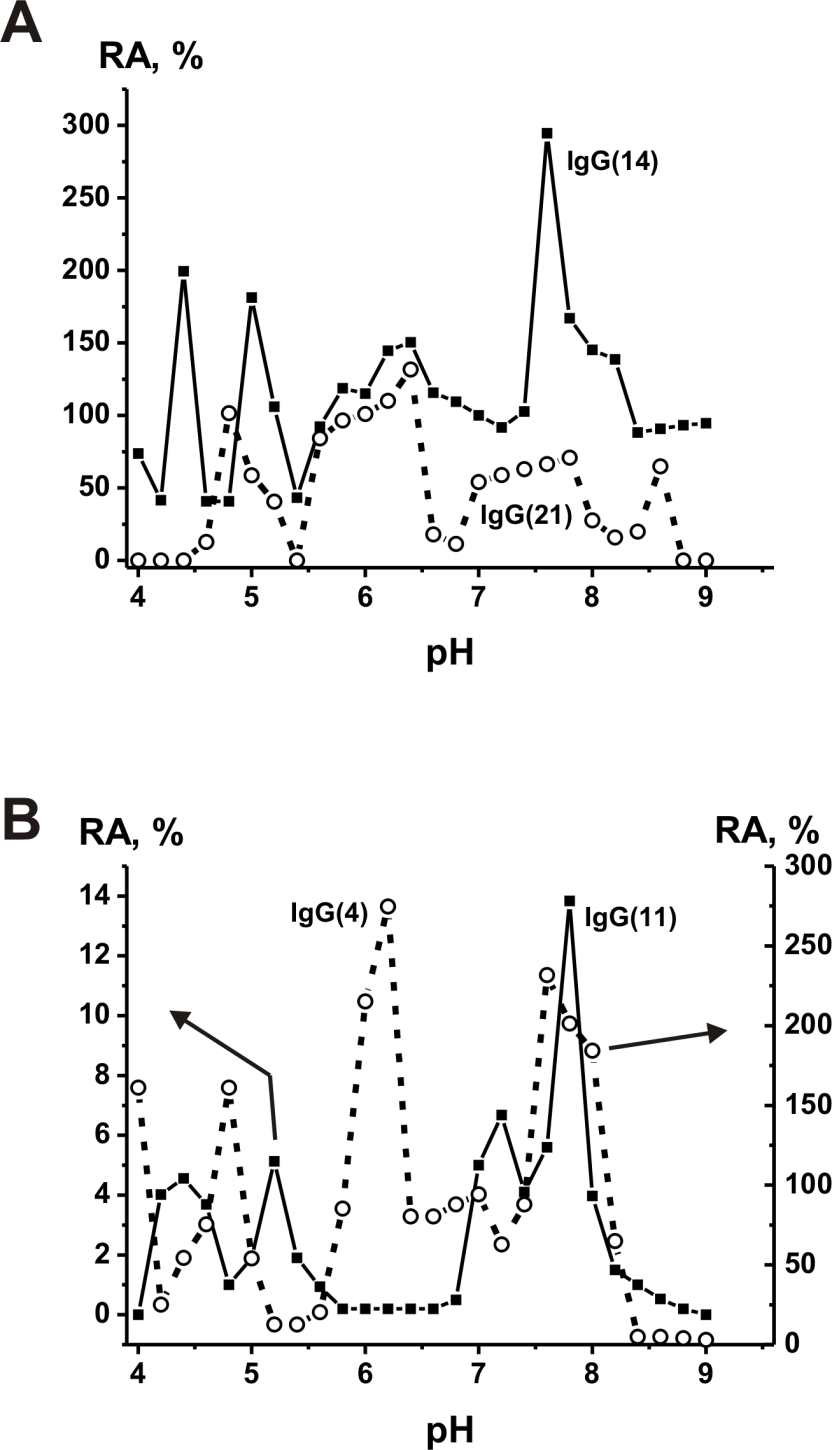
**Dependencies of the relative catalase activity of scz-IgGs on pH of the reaction mixture** (**A** and **B**). All relative activities of Abs before its preincubation were taken for 100%. The relative activity of IgG(14) at pH 7.0 was taken for 100%. The errors in the determination of initial rate from three experiments in each case did not exceed 7–10%. For details see Materials and Methods.

Various oxidoreductases have different pH optima, but each has only one pH optimum [67]. Human catalase pH optima are approximately 7.0 [84]. In contrast to all canonical enzymes, the pool of polyclonal Abs from the sera of patients with different diseases can contain many monoclonal abzymes; some preparations including IgGs with peroxidase activity can demonstrate up to 8 pronounced optima at pH range from 5 to 10 [40–45, 72, 74, 75]. We have analyzed pH optima for four non-dialyzed scz-IgGs (Fig 6). All scz-IgGs demonstrated several very well expressed pH optima at pH values from 4.0 to 9.5 (Fig 6). All scz-IgGs have increased catalase activity at pHs 5.5–6.5 and 7.5–8.5. IgG(14) and IgG(11) show two pH optima at pHs from 4.0 to 5.5, while IgG(4) and IgG(21) demonstrate in this region of pHs only one optimum at pHs approximately 4.5–4.7 (Fig 6). In contrast to human catalase, optimal pHs of four scz-IgGs are not equal to 7.0 (Fig 6). The maximal activity dependently of IgGs at different specific pHs is 2.4–3.0-fold higher than that at pH = 7.0.

## Discussion

As mentioned above, 83% of IgGs from healthy Wistar rats possess superoxide dismutase activity, while all preparations oxidized 3,3'-diaminobenzidine in the presence and the absence of hydrogen peroxide, but only 17% of IgG preparations possessed catalase activity [68].

We have demonstrated, that catalase activity is an intrinsic property of 33.3–36.4% IgGs derived from the sera of SCZ patients and healthy humans. Application of a set of strict criteria including analysis of catalase activity of human IgGs using detection after SDS-PAGE revealing the activity of F(ab)_2_ and Fab_2_ fragments rule out possible artefacts due to contaminants (Fig 2). The relative activity of F(ab)_2_ is significantly higher than that for F(ab) fragments (Fig 2D). It should be assumed that F(ab)_2_ can have a more stable spatial structure than the F(ab) fragments. Therefore there may be a more effective unfolding F(ab) fragments leading to a decrease in the catalytic activity of their active centers.

In contrast to rat IgGs [73–76], but similar to human IgGs with peroxidase activity [72] the sera of humans contain not only Me-dependent but also Me-independent subfractions of IgGs with catalase activity. The ratio of metal-dependent and independent catalase activity is individual for every IgG preparation and varies significantly before and after IgG dialysis.

It is known, that mammalian, bacterial, and plant peroxidases, oxidoreductases, oxidases and dismutases are more often dependent on one type of metal ions, more often Fe^2+^, Mn^2+^, or Ni^2+^ and only superoxide dismutases are Cu^2+^,Zn^2+^ dependent enzymes [31, 64, 65, 67]. In contrast to these canonical peroxidases and oxidoreductases, but in some extent similar to rat IgGs [73–75], specific metal-dependent subfractions of human IgGs with oxidoreductase and peroxidase activity are activated by Fe^2+^, Cu^2+^, and Mn^2+^ [72].

Activation of dialyzed IgGs by metals ions with variable valences may indicate the involvement of these metal ions directly in the catalytic center and decomposition of H_2_O_2_. Among the metal ions with a variable valence, the best activator of three dialyzed scz-IgGs is Co^2+^ (Figs 4 and 5), which previously was not found as a good activator of canonical oxidoreductases and IgGs with peroxidase and oxidoreductase activities. As one can see in Fig 4B, the dependence of the relative activity of IgG(12) on the concentration of CoCl_2_ has two linear segments, and there is no Ab inhibition or plateau up to 10 mM concentration of the salt. These data may indicate for the fact that IgG(12) contains a set of monoclonal catalase abzymes with a different affinity for Co^2+^ ions.

Dialyzed IgG(7) is also significantly activated by Mn^2+^ (Fig 5B). In the case of dialyzed IgG(4) several metal ions with variable valences can stimulate its catalase activity: Co^2+^ ≥ Mn^2+^ ≥ Fe^2+^ ≥ Ni^2+^. All of them can increase the IgG-dependent catalase activity. Interestingly, in contrast to canonical catalases, Fe^2+^ ions did not activate catalase activity of dialyzed IgG(12) and IgG (7) (Fig 5B and 5C), but increase the activity of IgG(4) (Fig 5A). Cu^2+^ did not activate catalase activity of three dialyzed scz-IgGs (Fig 4).

Zn^2+^, Ca^2+^, and Mg^2+^ ions cannot be cofactors of catalases stimulating degradation of H_2_O_2_, and they do not activate IgG(7) and IgG(12) (Fig 5B and 5C). At the same time, Zn^2+^, Ca^2+^, and Mg^2+^ ions with constant valence remarkably increase the activity of dialyzed IgG(4) (Fig 5A). However, Abs can contain multiple metal binding sites. Metal binding at a remote site (as opposed to the catalytic site) chelating Zn^2+^, Ca^2+^, or Mg^2^ may stabilize the conformation of IgG molecule, thereby altering catalytic activity of metal-dependent and metal-independent abzymes leading to increasing their activity. Interestingly, some oxidoreductases, like mammalian Cu, Zn dependent dismutases, contain only one Cu^2+^ ion that participates in the oxidation reaction directly. The other Zn^2+^ ion with a constant valence is necessary for activation of the process. Moreover, some oxidoreductases can use two different or identical metal ions with variable oxidation states, which participate in the oxidation reaction directly [85]. Therefore, one cannot exclude that the activation of dialyzed IgG(4) by Zn^2+^, Ca^2+^, and Mg^2+^ may be a consequence of participation of these ions in the direct activation of H_2_O_2_ reduction by metal ions with variable oxidation state.

It was interesting to analyze the effects of different metal ions on the catalase activity of non-dialyzed scz-IgGs containing intrinsically bound metal ions. Co^2+^ is the best activator of all three non-dialyzed scz-IgGs (Fig 5). Mn^2+^ ions significantly activate dialyzed IgG(7), slightly inhibit non-dialyzed IgG(7), and have no remarkable effects on the activity of dialyzed and non-dialyzed IgG(12), but activate IgG(4) before and after the dialysis. While Cu^2+^ ions cannot activate three dialyzed scz-IgGs, they significantly increase the catalase activity of non-dialyzed IgG(7) and IgG(12) but were inactive in the case of dialyzed and non-dialyzed IgG(4) (Fig 5). There is remarkable inhibition or at least the absence of pronounced effects of Ca^2+^, Ni^2+^, Fe^2+^, and Mn^2+^ in the case of non-dialyzed IgG(4), IgG(7), and IgG(12). Mg^2+^ activates only non-dialyzed IgG(4), while Cu^2+^ increases the activity only IgG(7) and IgG(12). Inhibition of the catalase activity of non-dialyzed scz-IgGs by the metal ions mentioned above may be a consequence the competition of these metal ions with optimal activator ions for scz-IgGs active centres. One cannot exclude that at physiological conditions Co^2+^and probably Mn^2+^ may be the most important metal ions stimulating catalase activity of abzymes in human blood. At the same time, some monoclonal molecules of total IgG pool can be activated by Fe^2+^ or Ni^2+^ ions (Fig 5), while non-dialyzed scz-IgGs may be activated by combinations of different metal ions. The data speak for an individual repertoire of catalase IgGs in the sera of SCZ patients and extreme diversity of these abzymes in their affinity to different metal ions and ability to activate catalase Abs.

It was shown, that all human canonical enzymes (including oxidoreductases) have one pronounced pH optimum. In contrast, polyclonal catalase scz-IgGs from the sera of individual patients demonstrate quite distinct pH dependencies (from four to five pH optima) within a wide range of pH values (4.5–9.5) (Fig 6). Thus, catalase scz-IgGs are very heterogeneous in their pH optima, activation by different metal ions and relative catalytic activity. Interestingly, these results correspond to the extreme diversity of peroxidase and oxidoreductase activities of rat and human IgGs [72–75] and the general situation concerning a very high diversity of polyclonal abzymes with various hydrolytic catalytic activities in the sera of autoimmune patients and human milk [40–45]. Polyclonal Abz with the nuclease, protease, ATP-, and polysaccharide-hydrolyzing activity can contain kappa or lambda light chains and possess high activity at various optimal pH values. Such antibodies can be activated by metal ions, can have many different net charges as well as *k*_*cat*_ and *K*_*m*_ values and have different substrate specificities [40–45].

In the case of artificial Abz catalysis, characterized by low reaction rates, *k*_*cat*_ values are approximately 10^2^–10^6^-fold lower comparing to corresponding classical enzymes with the same catalytic activity [39]. A similar situation was observed for natural abzymes from autoimmune patients [40–45]. There is only one example of natural abzymes with the specific catalytic activity comparable with the canonical enzyme; RNA-hydrolysing IgGs from 100 patients with MS usually demonstrated specific activities up to 10% but IgGs from four patients possessed 40–400% activity as of pancreatic RNase (100%) [86]. These IgGs were considered as an example of natural abzymes with the highest specific activity. Later it was shown, that the specific peroxidase and oxidoreductase activity of rat and human IgGs is high (10^2^–10^4^ min^-1^) approximately 2–4 orders of magnitude higher than the specific activities of most known artificial and natural abzymes [72–75].

It was shown that *k*_cat_ values of catalase reaction ((1.9–3.4)×10^5^ min^-1^) by non-dialyzed scz-IgG antibodies depend on hydrogen peroxide. Several reasons allow us to suggest that the specific activities of catalase scz-IgGs may be significantly higher than we have observed for the non-dialyzed preparations (Fig 3, Table 1). First, the specific activities of the analyzed Ab preparations were calculated using the total concentration of polyclonal IgGs. Also, as we have shown earlier, acidic shock purification stage during Abs isolation lead to a significant inactivation of Abs in all catalytic activities analysed [40–45]. Moreover, for comparison of relative activities of scz-IgGs and health-IgGs, we have used pH of reaction mixtures equal to 7.0, which is optimal for human catalase but not optimal for all Ab preparations used. For example, the transition from pH = 7 to different specific optimal pHs for various scz-IgGs leads to increase in the RAs 2.4–3.0-fold (Fig 6). An 1.3–3.0-fold increase in the relative activity was observed after addition to non-dialyzed scz-IgGs of 1.5 mM Co^2+^ (Fig 5). Increase in the Co^2+^ concentration to 10 mM can lead to increase in the activity approximately 5–7-fold (for example, Fig 4B).

The average RA of catalase activity of scz-IgGs is ~15.8-fold higher than that for healthy-IgGs. At the same time, the relative percent of healthy donors and SCZ patients, the sera of which contain catalase IgGs are comparable, 33.3 and 36.4%, respectively. On the one hand, one cannot exclude that the production of the catalase abzymes can occur in healthy and in sick individuals with an increased formation of Abs in total pools of auto-Abs. In this case, the specific processes occurring in schizophrenia patients can lead to an increased production of the pool of auto-Abs including abzymes with high catalase activity. It is believed that damage of brain cells membranes of schizophrenia patients, as a consequence induces the formation of autoantigens and autoantibodies. But the importance of immunological changes leading to the loss of tolerance to self-antigens in the genesis of schizophrenia was not established. It was shown, that human antibodies from the serum of autoimmune patients [40–45] and mouse DNase IgG [87] are early markers of autoimmune pathology. Also, it was shown that DNase activity is detectable even at the stage of pre-disease when there is no any visible markers of SLE or other autoimmune pathology. During the pre-disease concentration of protein in the urine, titres of anti-DNA antibodies are within the typical ranges for healthy mice. Antibodies with DNase activity were recently revealed in 80% of SCZ patients [23]. These data indicate that some schizophrenia patients may show signs of typical autoimmune processes to a certain extent. At the onset of autoimmune diseases (pre-disease condition), catalytic antibodies are usually presented by a single clone or at least a relatively narrow repertoire of Abzs with relatively low activities. In the case of chronic autoimmune pathology development, the repertoire of abzymes with different activities expands and Abzs with significantly higher RAs can be found. Therefore, it is possible that the accumulation of the abzymes with high catalase activity in patients with SCZ is a consequence of patient’s autoimmune processes usually leading to the common increase in the titres of auto-Abs and abzymes with various catalytic activities.

Human organisms are constantly exposed to oxidative stress and various toxic components. It was shown, that the sera of healthy humans and mammals contain abzymes with superoxide dismutase [88, 89], metal-dependent and independent peroxidase and oxidoreductase oxidizing various aromatic amines and phenols [72–75]. We revealed catalase activity of IgGs from the sera of healthy humans and SCZ patients. These data indicate that some Abz may form not only in patients with autoimmune diseases but also in healthy humans. Therefore, one cannot exclude that toxic, mutagenic and carcinogenic compounds falling into organisms of healthy humans may cause the production of abzymes with different oxidoreductase activities. Also, anti-idiotypic Abs against active centres of different enzymes can also possess catalytic activity [40–45]. It was shown that DNase abzymes of autoimmune patients present a "cocktail" of Abs directly to DNA and RNA and anti-idiotypic Abs against active centres of DNase I, DNase II, RNase and other enzymes hydrolysing nucleic acids. Thus, we cannot exclude the possibility of different oxidoreductase abzymes formation as anti-idiotypic Abs against canonical enzymes: catalase, glutathione peroxidase, superoxide dismutase oxidizing different substrates in parallel with Abs against mutagens and carcinogens. The immune system theoretically can produce up to 10^6^ different Ab variants in response to a single antigen; therefore, catalytic diversity of abzymes with oxidoreductase activities including catalase Abs is not surprising.

In addition to canonical mammalian superoxide dismutase, catalase glutathione peroxidase, and other oxidoreductases, abzymes with these activities can be considered as natural proteins for detoxification of reactive oxygen species, and destruction of toxic, carcinogenic, and mutagenic compounds. Thus, it is possible that human catalase IgGs can also have a protective biological function similar to that of classical catalase.

## Materials and methods

### Chemicals, donors, and patients

Most chemicals and proteins were from Sigma; the Superdex 200 HR 10/30 column was from GE Healthcare. Sera of 22 patients (20–61 yr old; average value 33.0 ± 7.4; 12 men and ten women) with clinically definite SCZ were used to study catalase abzymes (the total group includes 12 patients with positive and ten patients with negative symptoms of the disease). Patients with positive SCZ symptoms were characterized by different manifestations of psychosis: delusions, tactile, auditory, visual, olfactory, and gustatory hallucinations, disordered thoughts and speech [90]. Hallucinations were also typically related to the content of the delusional theme [91]. Negative symptoms are explained as deficits of normal emotional responses or other thought processes and are less responsive to medication [92]. Negative SCZ symptoms commonly include flat expressions or little emotion, poverty of speech, inability to experience pleasure, lack of desire to form a relationship, and lack of motivation. Negative symptoms appear to contribute more to the poor quality of life, functional ability, and the burden on others than positive symptoms do [93]. The SCZ diagnosis was confirmed and checked according to the PANSS (positive and negative syndrome scale) standard international psychometric criteria. Positive, negative, and general psychopathology parameters, and CGI (Clinical Global Impression) were verified. Doctors of Department of endogenous disorders of the Mental health research institute (Tomsk, Russia) provided us clinically verified diagnoses according to International Classification of Diseases, 10^th^ revision (ICD-10). It was shown by specialists, that 22 patients with schizophrenia had a negative history of typical systemic autoimmune or rheumatic diseases.

For comparison, we used the sera of 21 conditionally healthy donors having no history of rheumatologic, respiratory, autoimmune, gastrointestinal, reproductive, cardiovascular, or nervous system pathology. The blood sampling protocol conformed to the local human ethics committee guidelines (Ethics Committee of Mental Health Research Institute of Tomsk National Research Medical Center of the the Siberian division of Russian Academy of Sciences). The permission of the Institutional ethics committee specifically approving the study was obtained including written consent of healthy donors and patients to present blood serum for scientific purposes following Helsinki ethics committee guidelines.

### IgG purification

Electrophoretically and immunologically homogeneous IgGs from the sera of SCZ patients and healthy donors were obtained by affinity chromatography of the serum on protein G-Sepharose and fast protein liquid chromatography (FPLC) gel filtration similarly to [71, 72]. A protein G-Sepharose column (1 ml) equilibrated in buffer A (50 mM Tris-HCl, 150 mM NaCl, pH 7.5) was loaded with the blood serum. The column was washed with buffer A to zero optical density (A_280_). Non-specifically bound proteins were eluted with 5 ml of the same buffer containing 0.5 M NaCl and 1% Triton X-100. The column was washed to zero optical density with buffer A. The 0.1 M glycine-HCl (pH 2.6) was used to elute IgGs from the column, fractions were collected into cooled tubes containing 50 μl of 1 M Tris-HCl (pH 8.8). Finally, each fraction was dialyzed against 50 mM Tris-HCl (pH 7.5). The fraction of IgG corresponding to the central part of the IgG peak was concentrated and used in further purification. Blood serum IgG preparations were filtered through a syringe drive Millex (pore size diameter 0.2 μm) to protect from bacterial contamination. In all cases the concentration of IgGs was determined by the Bradford assay (three independent repeats) with a bovine serum albumin standard.

IgG fractions corresponding to central parts of peaks after protein G-Sepharose elution were incubated in 50 mM glycine-HCl (pH 2.6) at 25^o^ C for 30 min. Fractions of IgG were separated under "acid shock" conditions was on Superdex 200 HR 10/30 FPLC gel filtration column equilibrated with 50 mM Gly-HCl (pH 2.6) containing 0.3 M NaCl as described previously [71, 72]. Fractions obtained on Superdex 200 were collected, neutralized and sterilized as described above. After one week of storage at 4^o^ C for refolding after the "acid shock", the central part of IgG peaks was used in the activity assays as described below.

### Catalase activity assay

Measurement of the IgG catalase activity was carried out according to [76]. For an accurate estimation of peroxide concentration we have used its manganometric determination according to [94]. Found by us concentration of hydrogen peroxide within the error of the method (± 3%) was as indicated on the manufacturer's packaging.

Reaction mixtures contain 50 mM potassium phosphate (pH 7.0), 30 mM H_2_O_2_, (2.8–56)×10^−8^ M IgGs (F(ab) or F(ab)_2_ fragments of IgGs). Catalase activity was determined spectrophotometrically from a decrease of absorbance at 240 nm for 1–10 min at 25^o^ C caused by the disproportionation of hydrogen peroxide using spectrophotometer Varian Cary 50 UV-VIS (Agilent) according to [76, 94]. All measurements (initial rates) were taken within the linear regions of the time courses and linear part of relative activity (RA) dependence upon IgG concentration. This approach allowed a normalization of the relative activity, like in the case of determination of the specific activity of enzymes, to any standard condition. For the calculation of the activity, the molar extinction coefficient of hydrogen peroxide (ε = 81 M^-1^cm^-1^) was used [95]. The measured relative activity of IgGs was normalized to standard conditions (mM H_2_O_2_ / min/mg IgGs).

The effects of metal ions on catalase activity of IgGs were analyzed after extensive dialysis against 20 mM Na-phosphate containing 0.1 M EDTA for 24 h at 4°C, then three times against 20 mM Na-phosphate for seven hours; all step of dialysis were performed in the vessels containing dialyze sack with a 5 ml of Chelex. IgGs dialyzed against EDTA were subjected to the second dialysis similarly to the fist dialysis but against 20 mM Na-phosphate solution, which was passed through 5-ml Chelex columns.

In some cases, CuCl_2_, FeSO_4_, MnCl_2_, NiCl_2_, CoCl_2_, and ZnCl_2_ (each at 0.1–5.0 mM) were added to reaction mixtures in the case of dialyzed and non-dialyzed IgGs. Also in parallel experiments, 0.1 M EDTA was added directly to the reaction mixtures in the case of non-dialyzed IgGs. The relative activity in the presence of Me^2+^-ions was assayed as described above using a standard reaction mixture; the activities of dialyzed Abs were compared with those for non-dialyzed IgGs. In the case of Me^2+^ effect analysis, the control mixtures contained the appropriate salts in required concentrations.

Thermal stability of IgGs was analyzed by preincubation of antibodies (1.8 mg/ml) in 50 mM potassium phosphate (pH 7.0) for 10 min at different temperatures (25-100^o^ C) and then 5 μl aliquots of the reaction mixture were added to 100 μl of standard mixture for estimation of catalase activity at 25^o^ C.

pH dependencies were analyzed using different buffer systems (20 mM): Na-phosphate-citrate (pH 4.0–6.0), Na-phosphate (pH 6.0–8.0), Tris-HCl (pH 8.0–9.5).

### SDS-PAGE analysis of catalase activity

SDS-PAGE analysis of IgG under reducing and non-reducing conditions was performed in 4–15% gradient gels (0.1% SDS) in the Laemmli system with silver staining (7–10 μg/protein per band) as in [71–75]. SDS-PAGE analysis of catalase activity of IgGs was performed similarly to studies of rat and human abzymes with oxidoreductase activities [71–75] before and after Abs treatment using 50 mM Tris-HCl (pH 7.5) containing 0.1% SDS and 40 mM DTT for 10 min at 30^o^ C. The enzymatic activity after SDS-PAGE was restored: SDS removing by incubating the gel for one hour at the room temperature with 4 M urea, and then in 10 changes (5–7 minutes) of milliQ H2O. The gel was washed five times with Na-phosphate pH 7.0 buffer. Then 3-4-mm cross-sections of longitudinal slices of the gel were cut out and incubated with 50 μl of Na-phosphate (pH 7.0) for five days at 4°C to allow protein refolding and elution from the gel. The eluates were used in the activity assay as described above. Parallel longitudinal gel slices were used to detect the position of IgG in the gel by Coomassie staining.

### Preparation of Fab and F(ab)_2_ fragments

The fraction of IgG_mix_ (equal amounts of IgGs from the sera of 22 SCZ patients) was cleaved with papain according to [96]. Fab fragments were purified by the affinity chromatography on protein A-Sepharose. The fraction not bound by protein A-Sepharose and appeared in flow contained Fab fragments and papain. Papain and Fab fraction was further separated by ion-exchange chromatography on a CM-Trisacryl column (1 ml) equilibrated with 50 mM Tris-HCl, pH 7.5. The fraction was applied in the same buffer and eluted with a NaCl gradient concentration from 0 to 1 M in the same buffer. The fractions obtained on CM-Trisacryl were collected and dialyzed against 10 mM Tris-HCl (pH 7.5), as described above. The Fab preparations were electrophoretically homogeneous.

The same IgG_mix_ preparation was cleaved with pepsin to obtain F(ab)_2_ fragments. F(ab)_2_ fragments were purified by chromatography on protein A-Sepharose. The fraction not bound by protein A-Sepharose contained F(ab)_2_, products of partial digestion of Fc fragment, and pepsin, and was further fractionated by gel filtration on a Superdex 200 HR 10/30 column equilibrated with Tris-buffered saline (TBS). The F(ab)_2_ fragment fractions were collected and dialyzed against 10 mM Tris-HCl (pH 7.5), as described above. The F(ab)_2_ obtained were electrophoretically homogeneous.

### Estimation of the kinetic parameters

The values of apparent *K*_m_ and *V*_max_ (*k*_cat_) were calculated from the dependencies of *V* versus [H_2_O_2_] by least-squares non-linear fitting using Origin 8 software and presented as linear transformations using a Lineweaver-Burk plot [97]. Errors in the values were within 7–15%. Here the results of linearization are reported as the mean ± standard deviation of at least three independent experiments for each IgG sample.

### Statistical analysis

The results here reported as the mean and the standard deviation of at least three independent experiments for each IgG preparation. The criterion of Shapiro-Wilk Test was used to check the normality of distribution. The most of the sample sets did not fit the Gaussian distribution. The differences between different groups of IgG samples were analyzed by the Mann–Whitney test, the value *P* < 0.05 was considered statistically significant. Median (M) and interquartile ranges (IQR) were also estimated.

## Abbreviations

Abs: antibodies
Abzs: abzymes, or catalytically active antibodies
AI: autoimmune
CGI: Clinical global impression
DAB: 3,3'-diaminobenzidine
HSA: human serum albumin
HRP: horseradish peroxidase
EDTA: ethylenediaminetetraacetic acid
IQR: interquartile ranges
LDH: lactate dehydrogenase
M: median
Me^2+^: any ions of divalent metals
SDS-PAGE: SDS—polyacrylamide gel electrophoresis
pIgGs: polyclonal IgGs
PANSS: standard international psychometric criteria (the positive and negative syndrome scale)
TG: thyroglobulin
SRXRF: Synchrotron radiation X-ray fluorescence
RA: relative activity.
